# Increased susceptibility of irradiated mice to *Aspergillus fumigatus* infection via NLRP3/GSDMD pathway in pulmonary bronchial epithelia

**DOI:** 10.1186/s12964-022-00907-2

**Published:** 2022-06-27

**Authors:** Dong-Ming Wu, Miao He, Yang-Yang Zhao, Shi-Hua Deng, Teng Liu, Ting Zhang, Feng Zhang, Yuan-Yi Wang, Ying Xu

**Affiliations:** 1 School of Clinical Medicine, Chengdu Medical College, The First Affiliated Hospital of Chengdu Medical College, Chengdu, Sichuan People’s Republic of China; 2 School of Laboratory Medicine, Chengdu Medical College, Chengdu, Sichuan People’s Republic of China

**Keywords:** *Aspergillus fumigatus*, Pulmonary infection, Radiation exposure, Pyroptosis, NLRP3 Inflammasome

## Abstract

**Background:**

*Aspergillus fumigatus* infection is difficult to diagnose clinically and can develop into invasive pulmonary aspergillosis, which has a high fatality rate. The incidence of *Aspergillus fumigatus* infection has increased die to widespread application of radiotherapy technology. However, knowledge regarding *A. fumigatus* infection following radiation exposure is limited, and the underlying mechanism remains unclear. In this study, we established a mouse model to explore the effect of radiation on *A. fumigatus* infection and the associated mechanisms.

**Methods:**

In this study, a mouse model of *A. fumigatus* infection after radiation was established by irradiating with 5 Gy on the chest and instilling 5 × 10^7^/ml Aspergillus fumigatus conidia into trachea after 24 h to explore the effect and study its function and mechanism. Mice were compared among the following groups: normal controls (CON), radiation only (RA), infection only (Af), and radiation + infection (RA + Af). Staining analyses were used to detect infection and damage in lung tissues. Changes in protein and mRNA levels of pyroptosis-related molecules were assessed by western blot analysis and quantitative reverse transcription polymerase chain reaction, respectively. Protein concentrations in the serum and alveolar lavage fluid were also measured. An immunofluorescence colocalization analysis was performed to confirm that NLRP3 inflammasomes activated pyroptosis.

**Results:**

Radiation destroyed the pulmonary epithelial barrier and significantly increased the pulmonary fungal burden of *A. fumigatus*. The active end of caspase-1 and gasdermin D (GSDMD) were highly expressed even after infection. Release of interleukin-18 (IL-18) and interleukin-1β (IL-1β) provided further evidence of pyroptosis. *NLRP3* knockout inhibited pyroptosis, which effectively attenuated damage to the pulmonary epithelial barrier and reduced the burden of *A. fumigatus*.

**Conclusions:**

Our findings indicated that the activation of NLRP3 inflammasomes following radiation exposure increased susceptibility to *A. fumigatus* infection. Due to pyroptosis in lung epithelial cells, it resulted in the destruction of the lung epithelial barrier and further damage to lung tissue. Moreover, we found that NLRP3 knockout effectively inhibited the pyroptosis and reducing susceptibility to *A. fumigatus* infection and further lung damage. Overall, our results suggest that NLRP3/GSDMD pathway mediated-pyroptosis in the lungs may be a key event in this process and provide new insights into the underlying mechanism of infection.

**Video abstract**

**Supplementary Information:**

The online version contains supplementary material available at 10.1186/s12964-022-00907-2.

## Background

Increasing evidence suggests that radiation exposure can cause significant decreases in the number of peripheral immune cells and releases pro-inflammatory factors, which weaken the host immune system and render the host susceptible to various infectious diseases [1]. Although large numbers of *Aspergillus fumigatus* conidia are suspended in the air, individuals with normal immune system rarely develop pulmonary aspergillosis owing to tolerance developed from lifelong exposure [2, 3]. However, in those with lung damage or a compromised immune system, *A. fumigatus* conidia can colonize the lungs and even cause invasive pulmonary aspergillosis, especially in patients receiving radiotherapy [4]. Invasive pulmonary aspergillosis is associated with an extremely high mortality rate, posing a tremendous challenge to public health. However, studies concerning the mechanism underlying *A. fumigatus* infection after radiation exposure are limited.

Radiation can induce a loss of pulmonary barrier function by destroying epithelial and endothelial cells [5]. Epithelial cells establish close contact with their neighbours through intercellular junction complexes (i.e., tight junctions and adherens junctions). Zonula occludens 1 (ZO-1) is an important component of tight junctions and decreases in ZO-1 expression levels play a key role in the injury and increased permeability of a variety of epithelial tissues [6]. Cadherins are components of adherens junctions that mediate cell–cell adhesion. Epithelial cadherin (E-cadherin) mediates adhesion between adjacent cells [7]. Disruption of and failure to reconstruct the pulmonary epithelial cell barrier can lead to catastrophic consequences. Once this barrier has been damaged, a large amount of blood exudates and inflammatory cells accumulate in the alveolar cavity, resulting in the aggregation of numerous fibroblasts that subsequently differentiate into myofibroblasts [8]. The bronchial epithelial cells seem to play a crucial role in the innate immune response against *Aspergillus*, particularly in preventing bronchial colonization. The high prevalence of bronchial *Aspergillus* colonization in patients with cystic fibrosis highlights this phenomenon [9, 10].

In recent years, pyroptosis, a distinct form of programmed cell death that differs from apoptosis, has been widely speculated as the underlying mechanism of radiation injury [11]. This process is associated with the occurrence and development of various diseases, including radiation-induced pneumonia, oral mucositis, and skin lesions [12–14]. Nucleotide-binding oligomerization domain-like receptors (NLRs) play an important role in pyroptosis and can identify pathogen-related molecular patterns or damage-related molecules [15]. NLRP3 can bind with the adaptor protein apoptosis-associated speck-like protein containing a caspase recruitment domain (ASC) and cysteine protease-1 (caspase-1) to form a dense protein complex (nucleotide-binding oligomerization domain-like receptors-ASC-caspase-1). Formation of this complex, which is called an inflammasome, leads to caspase-1 cleavage. The resulting activated form of caspase-1 can promote the processing, maturation, and secretion of the inflammatory cytokines interleukin (IL)-Iβ and IL-18 [16]. Research has indicated that radiation can promote the activation of NLRP3 inflammasomes, which can then mediate pyroptosis [17], and our preliminary evidence suggests that the NLRP3 inflammasome plays an important role in the regulation of radiation damage [18].

In this study, we aimed to explore the effect of radiation on *A. fumigatus* infection and the mechanisms that may be involved in this phenomenon using a mouse model. Based on the potential role of NLRP3 inflammasomes in radiation damage, we hypothesized that radiation would lead to the destruction of the pulmonary epithelial barrier via NLRP3-mediated pyroptosis, thereby increasing susceptibility to *A. fumigatus* infection.

## Methods

### Animals

Wild-type and NLRP3^−/−^ mice on a C57BL/6 J background were purchased from Beijing Weishanglide Biotechnology Co., Ltd (Beijing, China). Female C57BL/6 mice (6–8 weeks old, weighing 18–20 g) were used in our study. And all mice were raised in specific pathogen-free animal housing and provided with sterile food and water. All animal procedures were approved by the Animal Policy and Welfare Committee of Chengdu Medical College (Approval Number: CDYXY-2019–36). All of animal experiments were repeated 3 times independently, with 8 mice in each group.

### Animal irradiation

Mice were anesthetized with an intraperitoneal injection of 3.5% chloral hydrate (0.1 mL/10 g body weight); placed on a platform; and exposed to chest irradiation at 5, 10 or 20 Gy using X-RAD 160–225 instrument (Precision X-Ray, Inc., Branford, CT, USA; filter: 2 mm AI; 42 cm, 225 kV/s, 12.4 mA, 2.0 Gy/min).

### Preparation of A. fumigatus

The *A. fumigatus* strain used in this study was clinically isolated from a patient diagnosed with pulmonary *A. fumigatus* infection and cultivated at 37 °C for 3 d on Sabouraud dextrose agar. The collected conidia were washed, suspended in phosphate buffered saline supplemented with 0.1% Tween 20 (resuspension buffer), and separated from the mycelia using gauze. *A. fumigatus* conidia were counted microscopically and further confirmed using a haemocytometer. The resuspension buffer was centrifuged to obtain a precipitate containing *A. fumigatus* conidia, which were subsequently resuspended in phosphate buffered saline at a concentration of 5 × 10^7^ conidia/ml.

### In vivo A. fumigatus infection

Mice were mildly anaesthetized with 3.5% chloral hydrate and administered 50 μL of 5 × 10^7^ conidia/ml viable conidia via the intratracheal route while being maintained in an upright position. Within 1 or 2 h, the mice recovered completely. Clinical manifestations and weight changes in each group were recorded daily. The overall condition of the mice was assessed every 8 h after *A. fumigatus* infection, and body weight was measured every morning. At specific periods after infection, lung tissues were harvested from the mice, and the number of log_10_ colony-forming units (CFUs) per lung was evaluated to assess the fungal burden.

### Tissue isolation and fungal burden assessment

Mice were divided into 4 groups (n = 8 per group), and weight was monitored daily. They were euthanized, and sera and bronchoalveolar lavage fluid (BALF) were collected. The mice were then quickly and carefully dissected on an ice plate, and the lungs were harvested and photographed. Left lungs were preserved in 10% formalin overnight at 4 °C, whereas right lungs were removed from the chest cavity, crushed with a tissue grinder, and immersed in 2 ml phosphate buffered saline to release the conidia. Primary homogenate dilutions were quantitatively cultured by serial dilution, plated in triplicate on Sabouraud dextrose agar plates, incubated at 37 °C for 24–48 h, and the number of CFUs per gram of tissue was enumerated. The infection rate was then calculated. Fungal burden in the lungs was determined by a quantitative CFU assay. At selected time points, post infection lungs were aseptically removed and their wet masses determined using a precision balance.

### Antibodies

The following primary antibodies were used for western blotting: anti-ASC (ab180799), anti-caspase-1 (ab179515), anti-NLRP3 (ab263899), anti-gasdermin D (ab219800), anti-IL-1β (ab254360), anti-IL-18 (ab223293) (all purchased from Abcam, Cambridge, MA, USA), anti-F4/80 (28463–1-AP), anti-ZO-1 (21773–1-AP), anti-E-cadherin (20874–1-AP), and anti-AIM2 (66902–1-Ig) (all purchased from Proteintech). FITC goat anti-mouse IgG (H + L) and Cy3 goat anti-rabbit IgG (H + L) (A0516) were purchased from Beyotime Biotechnology (Haimen, China) and used for immunofluorescence analyses.

### Pathological staining and score

To evaluate the degree of inflammatory cell infiltration, lung tissues were dehydrated via an ethanol gradient, cleared with xylene, embedded in paraffin, cut into 5-μm sections, and stained using a haematoxylin and eosin staining kit (Solarbio, Revetal, Norway). For each mouse, 10 fields of the left lung at 20 × magnification were examined. Scoring was performed by grading as follows: infiltration or aggregation of inflammatory cells in air space or vessel wall: 1 = only wall, 2 = few cells (1–5 cells) in air space, 3 = intermediate, 4 = severe (air space congested); interstitial congestion and hyaline membrane formation: 1 = normal lung, 2 = moderate (50% of lung section); haemorrhage: 0 = absent, 1 = present.

A glycogen periodic acid-Schiff/haematoxylin stain kit (G1218; Solarbio, Beijing, China) and Grocott’s methenamine silver stains (M052; GEFAN BIOTECHNOLOGY, Shanghai, China) were used to detect the presence of fungi. Lung injury was assessed using a one-step terminal deoxynucleotidyl transferase dUTP nick end labelling apoptosis assay kit (C1088; Beyotime Biotechnology, Haimen, China) in accordance with the manufacturer’s instructions. Images were captured at 20 × and 40 × magnification (XI 71 microscope, Olympus, Tokyo, Japan).

### Immunofluorescence (IFC) staining

The sections were dewaxed with xylene, dehydrated with gradient ethanol, washed with PBS, sealed with 5% bovine serum albumin for 30 min at 37 °C, and incubated overnight with antibodies against ASC or NLRP3 (1:200) at 4 °C. The sections were washed in PBS and incubated with secondary FITC/Cy3 goat anti-rabbit/mouse IgG (H + L) for 2 h at 37 °C, following which the nuclei were stained with 4′,6-diamino-2-phenylindole. Fluorescence images were captured at 20 × magnification.

### Immunohistochemistry (IHC)

The Splink Test Kit (ZSGB-BioTechnology, Beijing, China) was used for the IHC analysis. First, the sections were boiled in an antigen repair solution (citrate buffer, pH 6.0). After natural cooling, they were blocked with 5% normal goat serum for 15 min at 37 °C, incubated with primary antibodies (1:200) for 8–10 h at 4 °C, and washed with PBS. Subsequently, the sections were incubated with the corresponding secondary antibodies for 2 h. To visualise the immunocomplexes, diaminobenzidine was used as the chromogenic agent, and haematoxylin was used for counterstaining. For each antigen, images were obtained at 20 × and 40 × magnification (XI 71 Olympus).

### Enzyme-linked immunosorbent assays and lactate dehydrogenase release assay

Peripheral blood was collected from the mice and centrifuged at 3,000 rpm for 15 min. For each group, a portion of the serum was used to quantify IL-1β and IL-18 levels using enzyme-linked immunosorbent assay kits (2 M-KMLJM211201m and 2 M-KMLJM219439m, Nanjing Camilo biological engineering co.LTD) and the release levels of IL-6, IL-8, IL-10, TNF-α and myeloperoxidase (MPO) were used enzyme-linked immunosorbent assay kits (Ruixin Biotech), with the remaining sera stored at − 80 °C until further analysis. BALF was used to detect lactate dehydrogenase (LDH) release from the lungs using an LDH kit (Mibio Biotechnology).

### Quantitative real-time polymerase chain reaction (qRT-PCR) analysis

In accordance with the manufacturer's instructions, total RNA was extracted from spinal cord tissues using a total RNA extraction kit (Solarbio), and cDNA was synthesised using an iScript cDNA synthesis kit (Bio-Rad, Hercules, CA, USA). The mRNA levels of 18sRNA, IL-6, tumour necrosis factor-α, IL-8, IL-10, ASC, AIM2, NLRC4, and NLRP3 were detected via qRT-PCR using SYBR Green SuperMix (Bio-Rad). The gene primer sequence was provided in the Additional file 3: Methods and Materials. Relative expression levels were calculated using the 2^−ΔΔCT^ method, with β-actin as the internal control. Primers were synthesised by Shanghai Shenggong (Shanghai, China).

### Western blot assay

Lung tissues were lysed in precooled RIPA cleavage buffer (Beyotime Biotechnology) for 4 h. Protein concentration was determined using a bicinchoninic acid (BCA) kit (Beyotime Biotechnology). Briefly, 60 μg of total protein was separated via 12% polyacrylamide gel electrophoresis and transferred onto a polyvinylidene fluoride membrane. The membrane was blocked with 5% skim milk for 1 h at room temperature and incubated with primary antibodies (1:1,000) for 6–8 h at 4 °C. After washing with Tris-buffered saline/0.1% Tween 20, the membrane was incubated with the secondary antibodies (1:5,000) for 2 h at 37 °C. Bands were detected using chemiluminescent horseradish peroxidase substrate and quantitatively analysed using Quantum 5.2 software (Bio-Rad). The relative level of immune response was assessed as a grey value and standardised against the reference protein (glyceraldehyde 3-phosphate dehydrogenase) using ImageJ software.

### Co-immunoprecipitation

Lung tissues were lysed in RIPA buffer. Protein A + G agarose (P2055-10 mL, Beyotime, Shanghai, China) was used for immunoprecipitation according to the manufacturer’s instructions.

### Statistical analyses

Data were analysed via one-way analysis of variance using Prism 7.0 software (GraphPad, La Jolla, CA, USA). Tukey’s post hoc test was used for multiple comparisons. Log-rank test was used for curves. Data are expressed as mean ± standard deviation. Statistical significance was set at *P* < 0.05.

## Results

### Radiation increased susceptibility to A. fumigatus infection

**Fig. 1:**
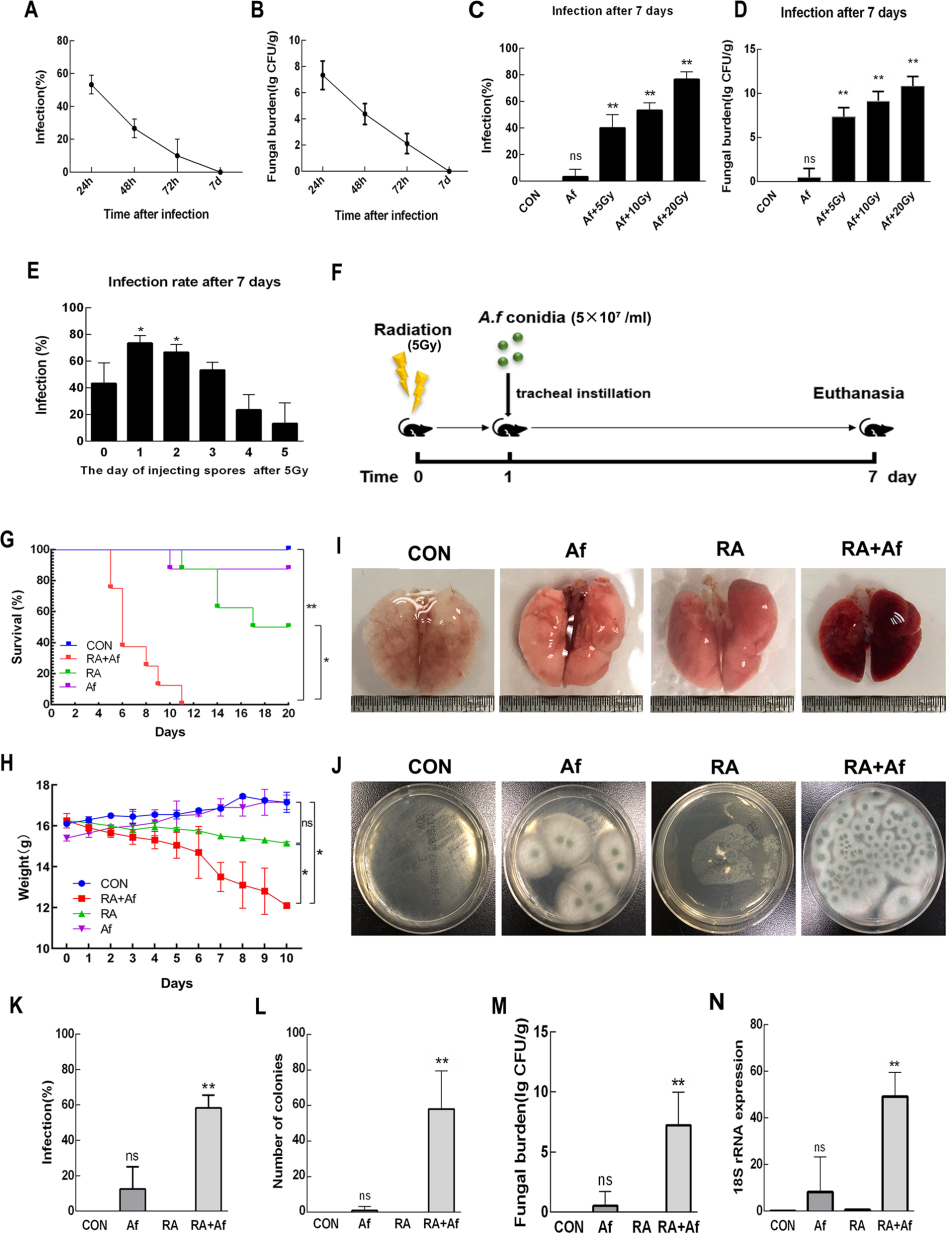
The establishment of a mouse model of post-radiation *Aspergillus fumigatus* infection. **a** Infection rates in C57 mice after intratracheal challenge by *A. fumigatus* conidia (5 × 10^7^/ml) were calculated at 24 h, 48 h, 72 h, and 7 Days. **b** Mice lung tissue homogenates were filtered and plated to measure log_10_ of colony-forming units (CFU). **c, d** The infection rate and fungal burden of different radiation doses observed after 7 days. **e** Rate of *A. fumigatus* infection when administered at different time points after irradiation. **f** Experiment process. **g, h** Survival status and changes in body weight in each group. **i** Representative images of lung tissues. **j** Lung tissue homogenates of each group were inoculated on Sabouraud dextrose agar, and **k** infection rates were calculated for each group. **l, m** The number of colonies and log_10_ of CFU in lung tissue homogenates. **n** Real-time polymerase chain reaction (PCR) analysis results for *A. fumigatus* and 18S rRNA levels in the lungs. Data are shown as the mean ± standard deviation (*n* = 8). *P < 0.05, **P < 0.01 versus the control group

Groups of C57 mice (n = 8/group) were administered a tracheal drip of *A. fumigatus* spore suspension (5 × 10^7^/mL), and fungal cultures of lung tissue homogenate were used to examine the infection status at different time points. Our findings indicated that both the infection rate and fungal load decreased over time. On Day 7 post-infection, *A. fumigatus* conidia had completely cleared (Fig. 1a-b). To explore the effect of radiation on *A. fumigatus* infection, mice were irradiated with different radiation doses (5–20 Gy) after anaesthesia, which was followed by tracheal instillation of conidia. Seven days after exposure to irradiation at 5 Gy, both the infection rate (Fig. 1c) and fungal burden (Fig. 1d) increased in fungal cultures of lung tissue homogenate. We also explored the impact of the time point at which conidia were administered. Conidia were injected at different time points (0–5 Days) after radiation, and the characteristics of lung infection were assessed 7 Days after treatment. Our results indicated that the infection rate started to increase when the conidia were administered on Days 1–2 after 5 Gy radiation exposure (Fig. 1e).

Based on the above experimental conditions, we explored the effects of radiation on *A. fumigatus* infection in the lungs. Mice were divided into four groups: CON: normal C57 mice; Af: infection only; RA: irradiation at 5 Gy only; RA + Af: irradiation at 5 Gy + injection of *A. fumigatus* conidia Day 1 after irradiation (Fig. 1f). Survival rate and body weight significantly reduced in the RA + Af group (Fig. 1g-h). Lung tissues exhibited clear congestion and were relatively small in size (Fig. 1i). Sabouraud dextrose agar cultures for *A. fumigatus* indicated that the rate of infection was higher in the RA + Af group and that a large amount of *A. fumigatus* had cultivated (Fig. 1j-k). On Day 7 after treatment, the number of fungal colonies and 18S rRNA expression levels remained high in the RA + Af group, while a very low index of infection was observed in the infection-only group (Fig. 1l-n).

### A. fumigatus conidia colonize and grow hyphae in the lungs of irradiated mice, which with clear inflammatory manifestations

**Fig. 2:**
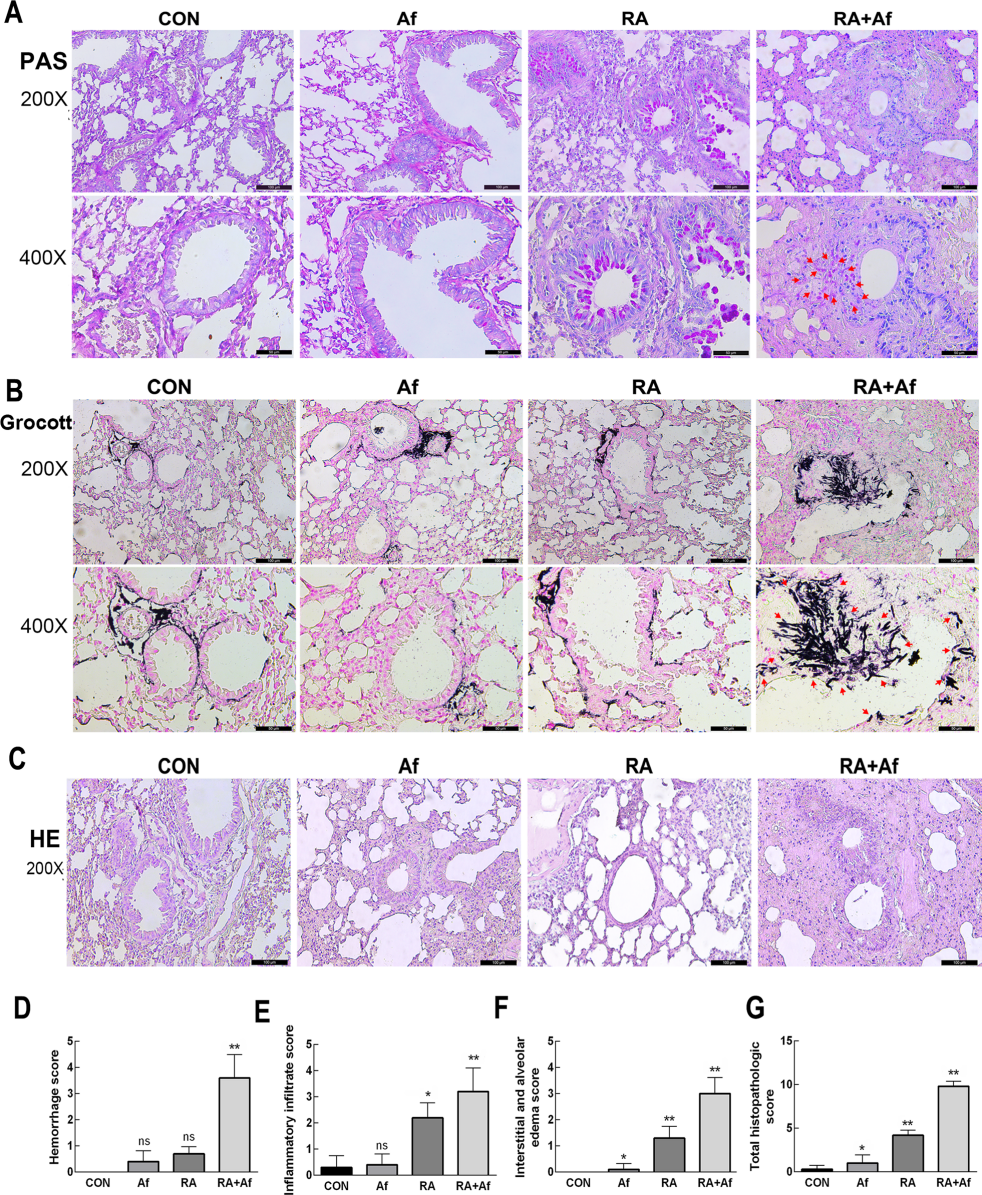
The degree of *A. fumigatus* lung infection in each treatment group. **a** Periodic acid-Schiff (PAS) and **b** Grocott's methenamine silver (GMS) staining results for the lungs. Scale bars: 100 μm and 50 μm. **c** Representative haematoxylin and eosin staining images showing inflammatory cell infiltration in the lungs. Scale bars: 100 μm. **d** Haemorrhage scores, **e** inflammatory infiltrate scores, **f** interstitial and alveolar oedema scores, and **g** total scores in each group. Data are shown as the mean ± standard deviation (*n* = 8). *P < 0.05, **P < 0.01 versus the control group

Periodic acid-Schiff and Grocott’s methenamine silver staining analyses indicated that, when compared with the infection-only group, the RA + Af group exhibited an increase number of *A. fumigatus* conidia invading the bronchi and surrounding lung parenchyma, and a large number of hyphae (Fig. 2a-b). Histopathological analysis of pulmonary tissues revealed leukocyte recruitment into the lungs in areas where the inflammatory infiltrate covered a large part of the pulmonary parenchyma structure, including the alveoli and perivascular regionsand this decreased in both Af and RA groups (Fig. 2c). Haemorrhage scores, interstitial and alveolar oedema, and inflammatory infiltrate levels were similar in the Af and RA groups. Moreover, mice in the RA + Af group exhibited more pronounced cellular infiltration, greater oedema, and more severe haemorrhage, which increased the total pathology scores (Fig. 2d-g). These data suggest that radiation affects susceptibility to *A. fumigatus* infection in the lungs and may exacerbate pulmonary injury. *A. fumigatus* may colonize the lungs of mice undergoing radiation treatment more readily than colonize those of non-irradiated mice, resulting in clear inflammatory responses.

### Radiation destroyed the bronchial epithelial barrier and induced more severe lung damage in irradiated mice infected with A. fumigatus

**Fig. 3:**
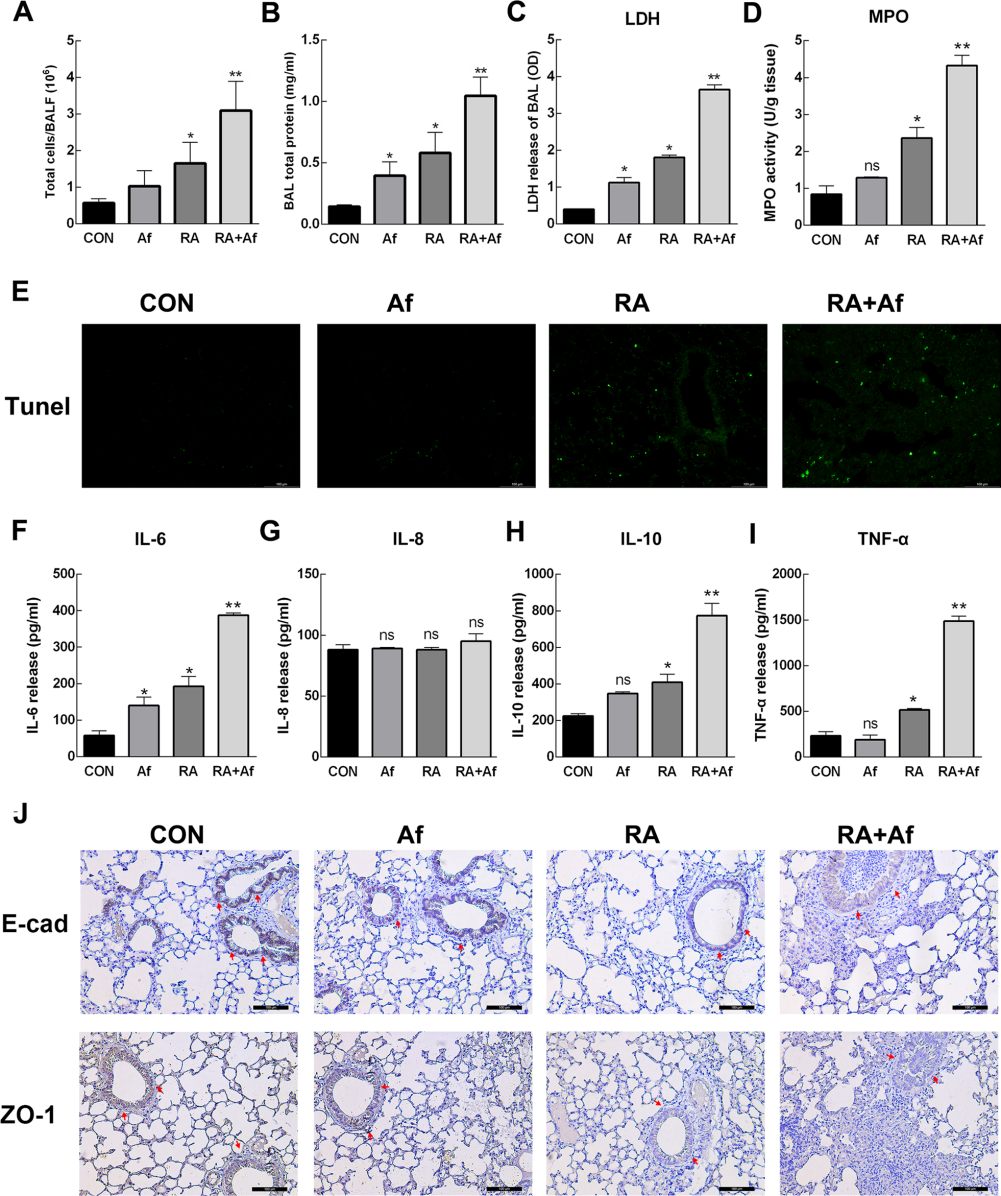
*A. fumigatus* infection after irradiation aggravated lung damage in mice. **a** Total leukocytes counts, **b** total protein levels, and **c** lactate dehydrogenase (LDH) release were assessed in the bronchoalveolar lavage fluid (BALF). **d** MPO release was assessed in the lung tissues. **e** Terminal deoxynucleotidyl transferase dUTP nick end labelling (TUNEL) staining in the lung tissues. Scale bars: 100 μm. **f–i** Elisa of interleukin 6 (IL-6), IL-8, IL-10, and tumour necrosis factor alpha (TNF-α) in serum. **j** Immunohistochemical analysis results of zonula occludens (ZO-1) and E-cadherin in the lung sections. Scale bars: 100 μm. Data are shown as the mean ± standard deviation (*n* = 8). *P < 0.05, **P < 0.01 versus the control group

To assess lung injury, we measured the number of leukocytes in the BALF by a Neubauer chamber. Following radiation, a large number of cells were recruited into lungs infected by *A. fumigatus* (Fig. 3a), and the bicinchoninic acid (BCA) and LDH assay results indicated that the BALF exhibited significantly increased protein levels (Fig. 3b) and high LDH levels (Fig. 3c). At the same time, the release level of MPO in RA + Af group was highest, that indicated there were a number of cells infiltrating into alveolar space (Fig. 3d). Terminal deoxynucleotidyl transferase dUTP nick end labelling staining revealed clear cell damage in bronchial epithelial cells (Fig. 3e). Significantly increased release of some inflammatory cytokines, including IL-6, IL-10 and tumor necrosis factor alpha in serum, excepting IL-8 (Fig. 3f-i). And their changes of mRNA expressions in lung tissue (Additional file 1: Fig. S1) are similar to that of serum. These results indicated that mice infected with *A. fumigatus* after radiation exhibited severe lung damage and inflammatory immune responses. As the integrity of the lung epithelial cell barrier is an important indicator of antifungal activity, we then evaluated levels of ZO-1 and E-cadherin expression to elucidate the mechanism underlying susceptibility to *A. fumigatus* infection after radiation treatment. Immunohistochemistry analysis revealed decreases in ZO-1 and E-cadherin expression in bronchial epithelial cells in both the RA and RA + Af groups, indicating that radiation had destroyed the bronchial epithelial barrier in these groups (Fig. 3j).

### Pyroptosis was continuously activated in the lungs of irradiated mice infected with A. fumigatus

**Fig. 4:**
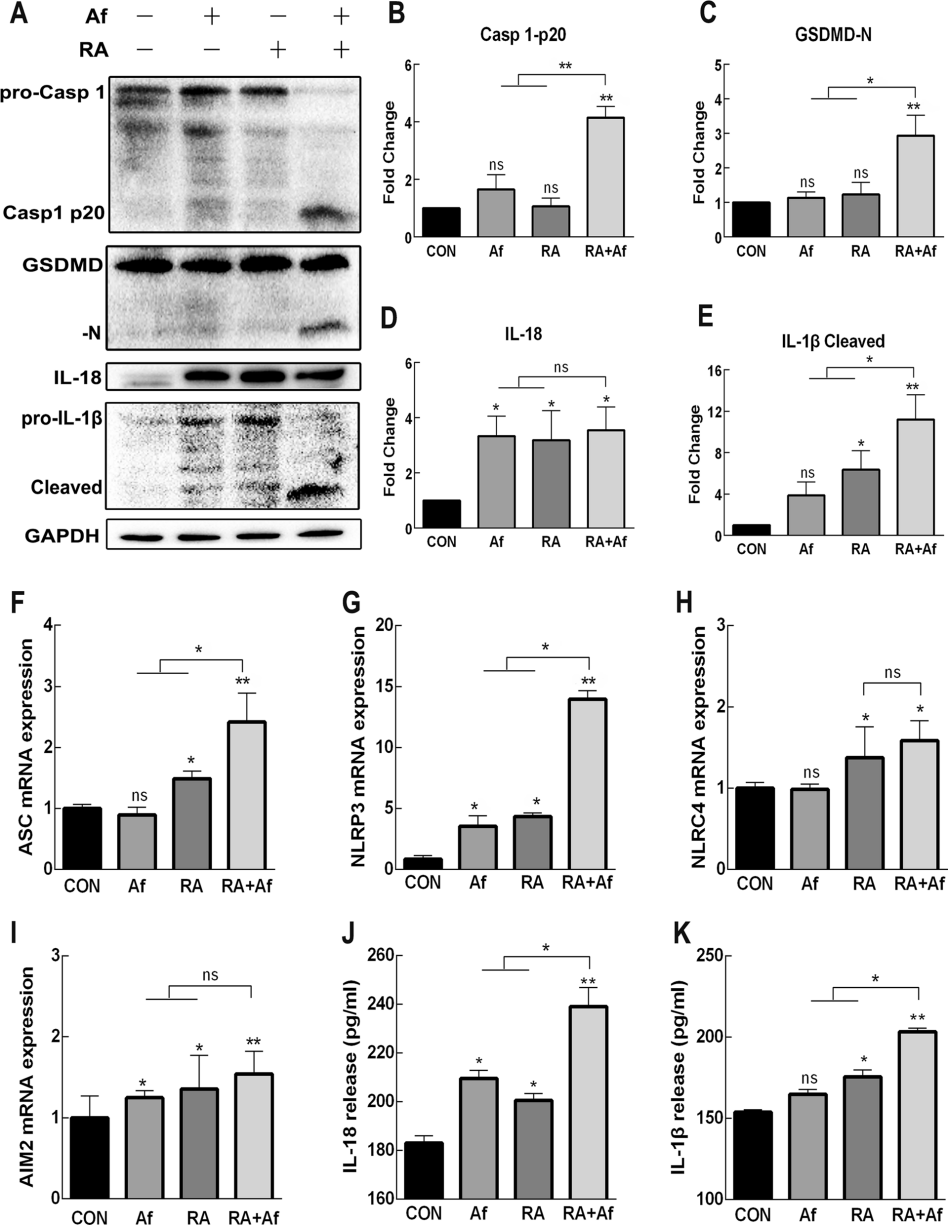
Pyroptosis is activated in the lung tissues of mice infected with *A. fumigatus* after irradiation. **a** Representative western blot images showing changes in the levels of caspase 1-p20, gasdermin D (GSDMD-N), interleukin 18 (IL-18), IL-1β cleaved, **b–e** and their statistical results. **f–i** mRNA expression of ASC, NLRP3, NLRC4, and AIM2. **j, k** Secretion of IL-18 and IL-1β in BALF. Data are shown as the mean ± standard deviation (*n* = 8). *P < 0.05, **P < 0.01 versus the control group

To explore whether pyroptosis occurred in irradiated mice, we first investigated changes in key proteins in the lungs over time after radiation. Our findings indicated that the active ends of caspase-1, gasdermin D (GSDMD), and IL-1β were significantly activated in 2–3 Days after radiation and disappeared on the 5th Day in the RA group, which was accompanied by an increase in IL-18 expression (Additional file 2: Fig. S2a). In the RA + Af group, these proteins were especially and continuously upregulated on the 7th Day after infection (Fig. 4a-e). We also measured mRNA levels of *ASC*, *NLRP3*, *NLRC4*, and *AIM2* and observed that the expression of *ASC* and *NLRP3* in the radiation-infected group were significantly higher than those in the three control groups were (Fig. 4f-i). The BALF of irradiated mice infected with *A. fumigatus* maintained high levels of IL-18 and IL-1β (Fig. 4j-k). Increased expression of these proteins was also observed the day following infection in both the RA and RA + Af groups (Additional file 2: Fig. S2b). These results suggested that pyroptosis occurred following radiation, with effects persisting in irradiated mice with *A. fumigatus* infection.

### NLRP3 inflammasomes are clearly activated in irradiated mice infected with A. fumigatus

**Fig. 5:**
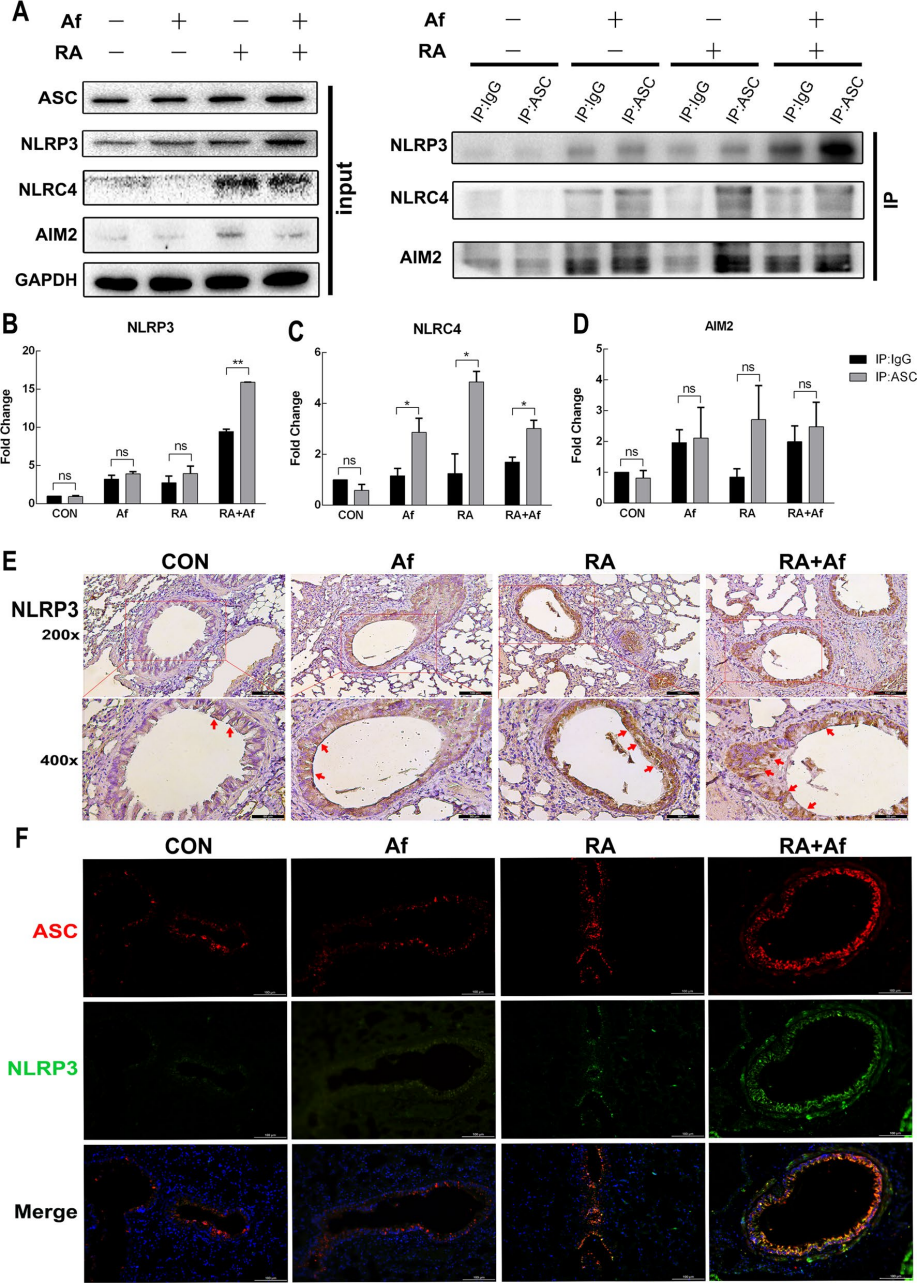
NLRP3 inflammasome activation in the lungs of mice infected with *A. fumigatus* after irradiation. **a–d** NLRP3, NLRC4 and AIM2 inflammasomes with CO-IP and the quantification graphs. (n = 8) *P < 0.05, **P < 0.01. **e** Immunohistochemical results for NLRP3 in lung tissues. Scale bars: 100 μm and 50 μm. **f** Representative immunohistochemistry double-staining images for ASC and NLRP3 in the lung sections. Red and green fluorescence indicate ASC and NLRP3, respectively. Scale bars: 100 μm

To explore further the mechanism underlying damage to the epithelial cell barrier in the lungs, we evaluated the expression of inflammasomes related to pyroptosis. Western blot analysis suggested that the treatment group had higher NLRP3 expression levels than the control group, and that the expression of NLRP3 increased significantly in the RA + Af group (Fig. 5a). The co-immunoprecipitation analyses detected protein binding with ASC, and significant colocalization of NLRP3 with ASC was observed in the RA + Af group (Fig. 5a-d). Immunohistochemical analysis of lung tissue indicated that NLRP3 was significantly upregulated in the bronchial epithelium in both RA and RA + Af groups (Fig. 5e). Furthermore, the binding between NLRP3 and ASC significantly increased in the bronchial epithelium of *A. fumigatus*-infected mice after irradiation (Fig. 5f). Overall, these results suggest that the NLRP3 inflammasome is strongly activated by NLRP3-ASC binding in pulmonary bronchial epithelia.

### *NLRP3*.^*−/−*^* mice exhibited reduced susceptibility to A. fumigatus infection and pulmonary injury after radiation exposure*

**Fig. 6:**
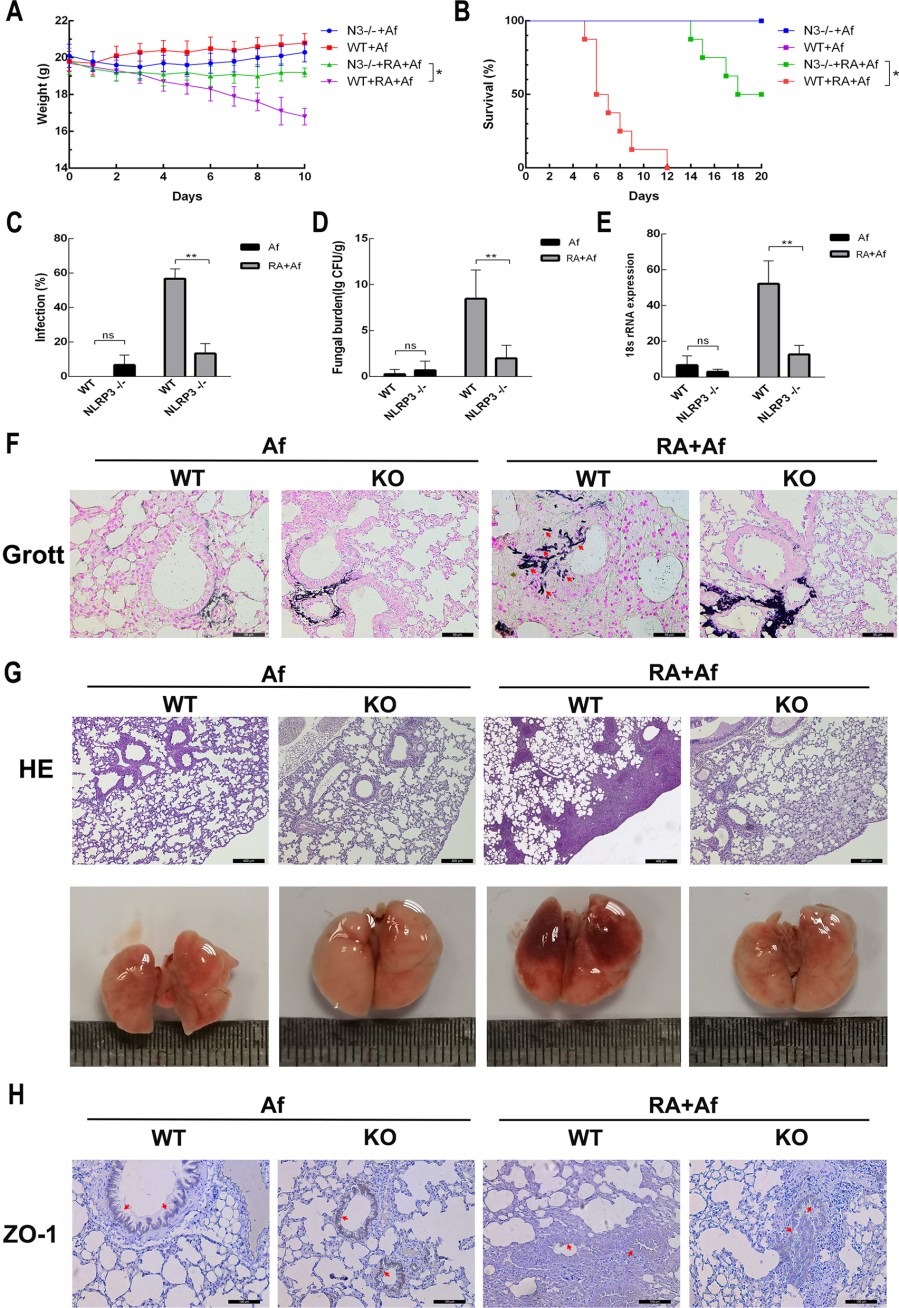
*NLRP3* knockout in mice reduces susceptibility to *A. fumigatus* infection and lung damage after radiation. **a** Survival status and **b** changes in body weight of wild-type (WT) and *NLRP3*^*−/−*^ mice in Af and RA + Af groups. **c** Infection rate, **d** fungal burden, and **e** expression of 18 s RNA of WT and *NLRP3*^*−/−*^ mice in Af and RA + Af groups. **f** Grocott's methenamine silver staining images of the lungs of WT and *NLRP3*^*−/−*^ mice in the Af and RA + Af groups. Scale bars: 50 μm. **g** Haematoxylin and eosin staining of lung tissue from WT and *NLRP3*^*−/−*^ mice in Af and RA + Af groups. Scale bars: 400 μm. **h** Immunohistochemical analysis results for ZO-1 of WT and *NLRP3*^*−/−*^ mice in the Af and RA + Af groups. Scale bars: 100 μm. Data are shown as the mean ± standard deviation (*n* = 8). *P < 0.05, **P < 0.01

To clarify further the role of NLRP3 in *A. fumigatus* infection after radiation, we assessed weight changes and rates of survival in *NLRP3*^−/−^ mice. *NLRP3* knockout effectively attenuated weight loss (Fig. 6a) and prolonged survival time (Fig. 6b) in irradiated mice with *A. fumigatus* infection. Furthermore, such knockout effectively reduced the susceptibility to *A. fumigatus* infection (Fig. 6c) and fungal burden (Fig. 6d-e). Grocott’s methenamine silver staining results indicated that colonization was also reduced in this group (Fig. 6f). These results suggest that *NLRP3* knockout helps reduce susceptibility to *A. fumigatus* after radiation in mice. In addition, haematoxylin and eosin staining of the lungs revealed that *NLRP3*^−/−^ mice exhibited decreased levels of inflammatory infiltration after radiation exposure and the lung tissues were clearly relieved of the congestion in *NLRP3*^−/−^ mice (Fig. 6g). *NLRP3* knockout also restored expression of ZO-1 in the bronchial epithelium (Fig. 6h). In summary, these data indicate that *NLRP3* knockout can help to reduce the infection rate and effectively alleviate lung damage in mice exposed to *A. fumigatus* after radiation.

### *NLRP3 knockout can effectively prevent pyroptosis in lung tissues with post-irradiation A. fumigatus infection *via* NLRP3/GSDMD pathway*

**Fig. 7:**
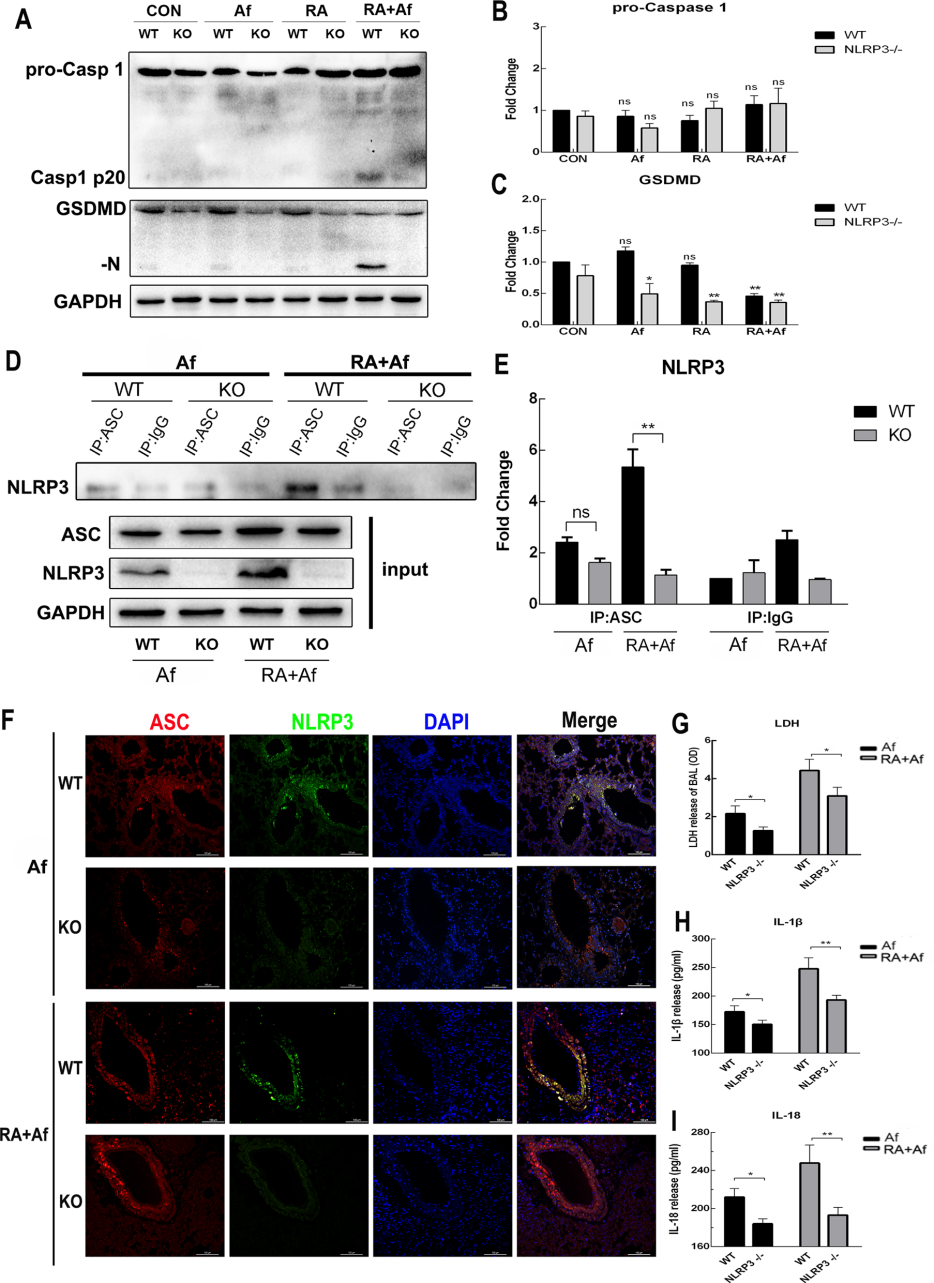
Following *NLRP3* knockout, pyroptosis is inhibited in mice infected with *A. fumigatus* after irradiation. **a–c** Representative western blot images showing changes in the levels of caspase 1-p20, and gasdermin D (GSDMD-N) in the lungs of wild-type (WT) and NLRP3^−/−^ mice and their gray value statistics. **d-e** The co-immunoprecipitation (CO-IP) results of ASC-NLRP3-inflammation and its gray value statistics. **f** Representative immunohistochemistry double-staining images of ASC and NLRP3 in the lung sections of WT and NLRP3^−/−^ mice. Red and green fluorescence indicate ASC and NLRP3, respectively. Scale bars: 100 μm. **g–i** Levels of lactate dehydrogenase (LDH), interleukin 18 (IL-18), and IL-1β in the bronchoalveolar lavage fluid (BALF) of WT and *NLRP3*^*−/−*^ mice in Af and RA + Af groups. Data are shown as the mean ± standard deviation (*n* = 5). *P < 0.05, **P < 0.01

We further explored the relationship between post-irradiation *A. fumigatus* infection and pyroptosis. Western blotting indicated that protein level of GSDMD decreased in all *NLRP3-/-* groups which was no difference for caspase-1, and that caspase-1-cleaved and GSDMD-N had not been activated after *NLRP3* knockout (Fig. 7a-c). Binding of ASC-NLRP3 protein was also decreased in the RA + Af group of *NLRP3*^*−/−*^ mice (Fig. 7d-e), which indicated that the activation of the NLRP3 inflammasome was obviously inhibited. Changes in the immunofluorescence double-staining analysis showed that the co-localization of NLRP3 and ASC was significantly reduced, indicating that the activation of the NLRP3 inflammasomes in *NLRP3*^−/−^ mice was reduced (Fig. 7f). Moreover, when compared with wild type mice, mice with *NLRP3* knockout exhibited significantly reduced release of LDH, based on an analysis of the BALF (Fig. 7g), as well as significant decreases in the secretion of IL-18 and IL-1β (Fig. 7h-i). These results indicate that *NLRP3* can regulate the initiation of pyroptosis in mice with *Aspergillus* infection after radiation via NLRP3/GSDMD pathway.

## Discussion

In this study, we examined the effect of radiation on *A. fumigatus* infection and the associated mechanisms using a mouse model. We observed that, after *A. fumigatus* lung infection, irradiated mice had a higher mortality rate and a significantly reduced ability for spore clearance. Histological analyses revealed increased lung inflammation/damage and reduced levels of tight junction proteins in lung epithelial cells, indicating barrier damage. Moreover, irradiated mice infected with *A. fumigatus* exhibited upregulation of NLRP3 in bronchial epithelia and activation of the NLRP3–ASC inflammasome. The cleaved end of caspase-1, which activates GSDMD to induce pyroptosis, was detected in the lungs of irradiated mice infected with *A. fumigatus*, along with IL-18 and IL-1β release. These results suggest that exposure to radiation leads to pyroptosis-induced damage in the lung epithelial cell barrier, in turn increasing susceptibility to *A. fumigatus* infection and accelerating lung injury, in which NLRP3 plays a key role.

With the widespread application of radiotherapy technology, lung infection has become a common complication, and the incidence of *A. fumigatus* infection has increased [19, 20]. Previous studies have shown that radiation damage leads to impaired lung function, which can reduce the clearance of *A. meristems* and increase the risk of IPA [21]. In this study, we established a C57 mouse model of post-radiation *A. fumigatus* infection to analyse susceptibility to infection and lung damage when compared with that in mice subjected to infection only. Our findings indicated that mice in the infection-only group could eliminate the infection within approximately 1 week after the invasion of *A. fumigatus*, and that the infection rate was lower in this group than in irradiated mice. In contrast, irradiation was associated with increased susceptibility to *A. fumigatus* infection and a decreased ability to clear fungal conidia. Moreover, the lung fungal load significantly increased as the radiation dose increased.

The spore concentration (5 × 10^7^/mL) used for *A. fumigatus* challenges was based on previous studies. This concentration has been used to assess the mortality and innate and adaptive immunities of animals that are not double immunosuppressed by cyclophosphamide and cortisone acetate [22, 23]. Our experiment neither focused on transient neutropenia nor used immunosuppressive drugs such as cyclophosphamide and cortisone acetate, as we speculated that inducing immunosuppression before infection may exaggerate the effect of radiation damage. Our experimental data clearly support the notion that radiation damage reduces lung clearance in immunized mice with *A. fumigatus* infection.

Data on the role of bronchial epithelial cells in anti-*Aspergillus* defense remain limited. However, bronchial epithelial cells play a crucial role in the innate immune response against *Aspergillus*, particularly in preventing bronchial colonization. Bronchial *Aspergillus* colonization, whose role in the subsequent development of IPA remains controversial, may have deleterious consequences, as it is the starting point for *Aspergillus* bronchitis and immuno-allergic forms [24, 25]. Lung epithelial cells act as a barrier to protect the respiratory system against various toxins and play an important role in defense and anti-infection function [7]. Located on the apical surface adjacent to the sidewall of alveolar epithelial cells, ZO-1 and E-cadherin facilitate the connections between cell membranes and are critical to the integrity of the alveolar epithelial barrier [6, 26]. Our results indicated that levels of ZO-1 and E-cadherin expression in lung bronchial epithelial cells were significantly reduced after radiation treatment, suggesting that radiation impaired alveolar epithelial barrier function, thereby leading to increases in *A. fumigatus* colonization.

The NLRP3 inflammasome plays a key role in the occurrence and development of a variety of inflammatory diseases, such as asthma, idiopathic lung disease, and radiation-induced pneumonia [27, 28]. NLRP3-mediated pyroptosis is involved in the repair of radiation and immune damage [29, 30], and the associated signal transduction pathway plays an important role in fungal infections [31–34]. Therefore, the role of the NLRP3 inflammasome in disease development and progression is a double-edged sword. On one hand, moderate activation can induce a protective effect; on the other hand, excessive activation can lead to severe damage in the host. In this study, on Day-2 after irradiation, the rate of infection with *A. fumigatus* conidia was highest, and the expression levels of proteins related to NLRP3-mediated pyroptosis were high. On Day-7 after irradiation, levels of NLRP3 expression and pyroptosis decreased in the radiation-only group, but the RA + Af group exhibited significant activation of NLRP3 and pyroptosis. A previous study demonstrated that *A. fumigatus* can activate inflammasomes only in the hyphal state [35]. Consistent with that study, our results showed that the invasion of simple *A. fumigatus* conidia was not associated with NLRP3 activation or pyroptosis.

Therefore, we speculate that lung epithelial cells are damaged by radiation-induced pyroptosis, thereby increasing susceptibility to *A. fumigatus* infection and colonization. The colonizing pathogen further activates NLRP3 inflammasomes and the inflammatory cascade, thereby accelerating lung injury. Our rescue experiments in NLRP3^−/−^ mice provide further support for this hypothesis, but further cell-level experiments are needed to clarify the underlying mechanism.

## Conclusion

In summary, our findings indicate that the rate of *A. fumigatus* infection in the lungs of a host with strong immune ability increases after irradiation, which results in obvious lung damage. Radiation-induced pyroptosis may contribute to the observed damage in the lung epithelial barrier, in turn leading to *A. fumigatus* infection. Controlling the inflammatory response caused by NLRP3-mediated pyroptosis may effectively reduce the *A. fumigatus* infection rate and the development of lung damage. Our mechanistic experiments provide a basis for understanding the role of NLRP3 in the risk of IPA after radiation. However, studies of NLRP3-mediated pyroptosis are still in their infancy in the context of post-radiation *A. fumigatus* infection, necessitating further mechanistic studies.

## Supplementary Information

**Additional file 1. Figure S1**. (**A**-**D**)The mRNA expressions of IL-6, IL-8, IL-10 and tumor necrosis factor alpha in lung tissue.

**Additional file 2. Figure S2.** (**A**) Representative western blot images showing changes in the levels of caspase 1-p20, gasdermin D (GSDMD-N), interleukin 18 (IL-18), IL-1β cleaved for 5 days after radiation only. (**B**) Representative western blot images showing changes in the levels of NLRP3, ASC, caspase 1-p20, gasdermin D (GSDMD-N), interleukin 18 (IL-18), IL-1β cleaved on the day following infection treatment in each group.

**Additional file 3. Table 1.** The gene sequence primer of qRT-PCR.

## Data Availability

Not applicable.

## Abbreviations

Af: Infection only
RA: Radiaton only
ASC: Apoptosis-associated speck-like protein containing a caspase recruitment domain
BALF: Bronchoalveolar lavage fluid
Caspase-1: Cysteine protease-1
CFUs: Colony-forming units
CON: Normal C57 mice
E-cadherin: Epithelial cadherin
GSDMD: Gasdermin D
IL: Interleukin
LDH: Lactate dehydrogenase
RA: Irradiation at 5 Gy only
RA + Af: Irradiation at 5 Gy + injection of *A. fumigatus* conidia Day 1 after irradiation
ZO-1: Zonula occludens 1
