# Supplementary figures and images for: Increased susceptibility of irradiated mice to Aspergillus fumigatus infection via NLRP3/GSDMD pathway in pulmonary bronchial epithelia
Source: Cell Commun Signal. 2022 Jun 27;20:98. doi: 10.1186/s12964-022-00907-2 (PMC9238178; doi:10.1186/s12964-022-00907-2)

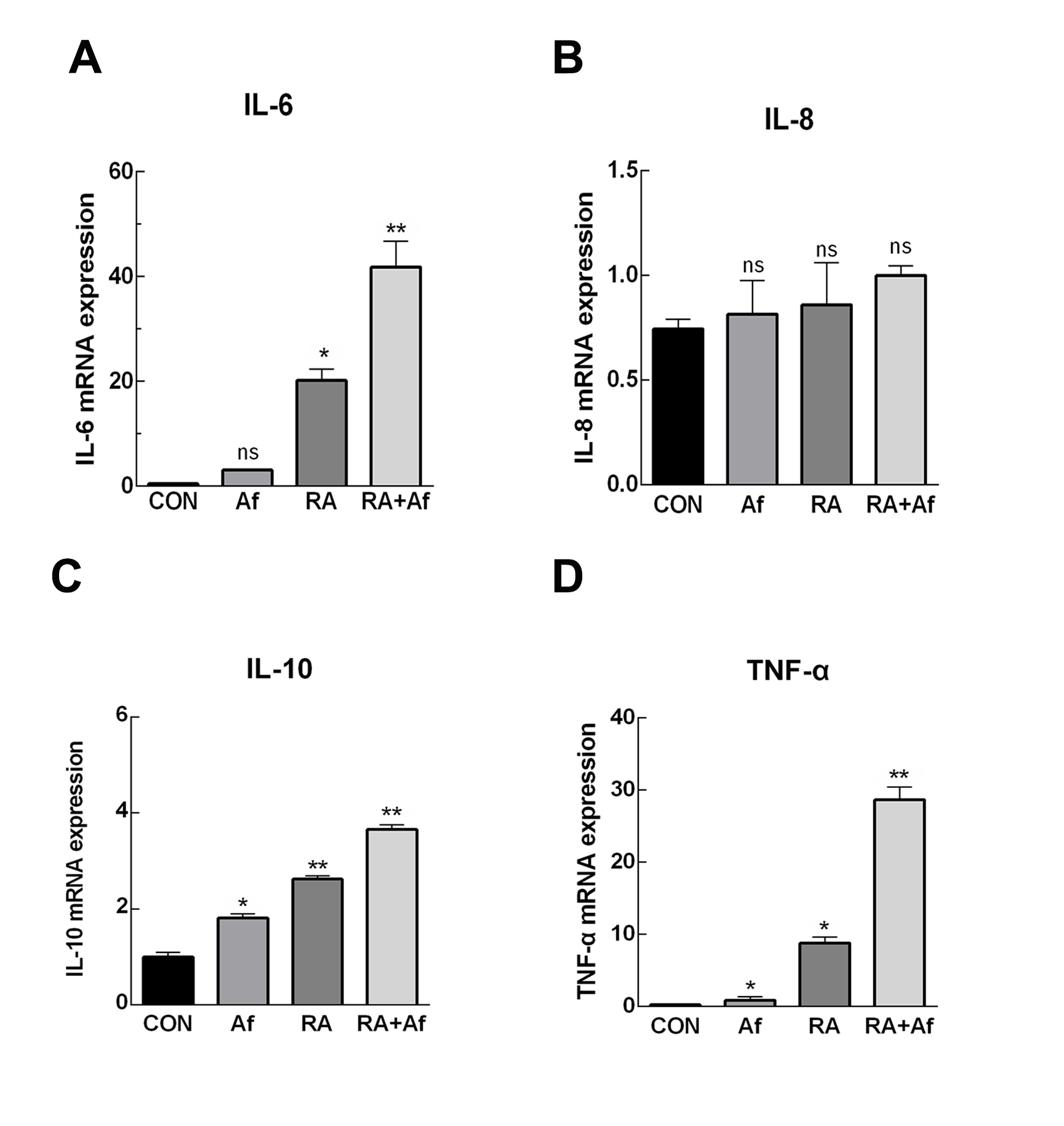

Supplement: Supplementary file 2 — Additional file 1. Figure S1. (A-D)The mRNA expressions of IL-6, IL-8, IL-10 and tumor necrosis factor alpha in lung tissue. [file 12964_2022_907_MOESM2_ESM.tif]

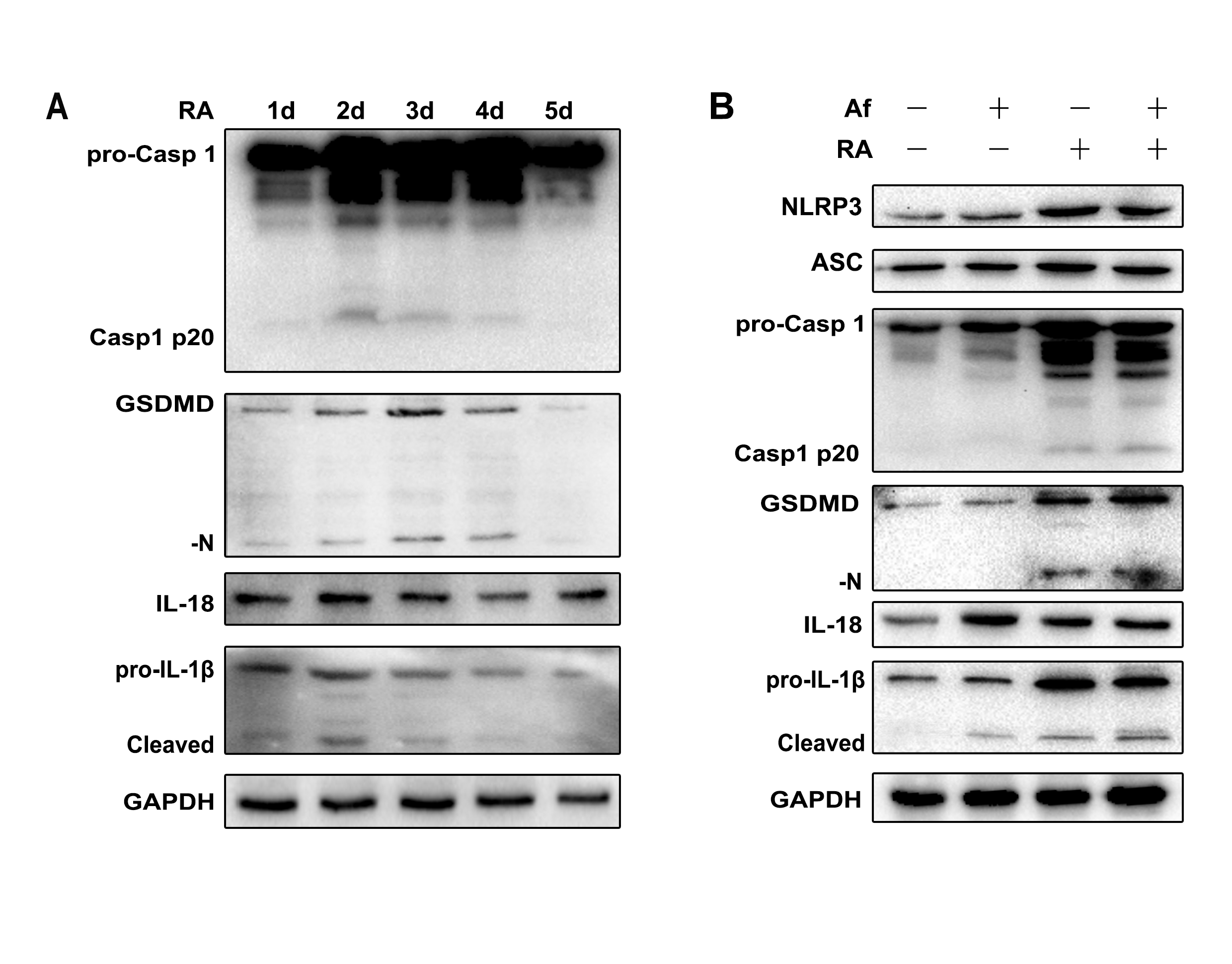

Supplement: Supplementary file 3 — Additional file 2. Figure S2. (A) Representative western blot images showing changes in the levels of caspase 1-p20, gasdermin D (GSDMD-N), interleukin 18 (IL-18), IL-1β cleaved for 5 days after radiation only. (B) Representative western blot images showing changes in the levels of NLRP3, ASC, caspase 1-p20, gasdermin D (GSDMD-N), interleukin 18 (IL-18), IL-1β cleaved on the day following infection treatment in each group. [file 12964_2022_907_MOESM3_ESM.tif]
