# Supplementary material for: Increased susceptibility of irradiated mice to Aspergillus fumigatus infection via NLRP3/GSDMD pathway in pulmonary bronchial epithelia
Source: Cell Commun Signal. 2022 Jun 27;20:98. doi: 10.1186/s12964-022-00907-2 (PMC9238178; doi:10.1186/s12964-022-00907-2)
Supplement: Supplementary file 4 — Additional file 3. Table 1. The gene sequence primer of qRT-PCR. [file 12964_2022_907_MOESM4_ESM.docx]

**Supplementary Methods and Materials**

**Supplementary Table 1**

Table 1 Gene primer sequence

| Gene | Primer sequence 5’→3’ |
| --- | --- |
| IL-6 | R：CTTGGGACTGATGCTGGTGACAAC |
|  | F：AGGTCTGTTGGGAGTGGTATCCTC |
| IL-8 | R：CATGGGTGAAGGCTACTGTTGGC |
|  | F：GCTTCATTGCCGGTGGAAATTCC |
| IL-10 | R：CACTGCTATGCTGCCTGCTCTTAC |
|  | F：TGGGAAGTGGGTGCAGTTATTGTC |
| TNF-α | R：TCTACTGAACTTCGGGGTGATCGG |
|  | F：GTGGTTTGTGAGTGTGAGGGTCTG |
| 18s rRNA | R：GGCCCTTAAATAGCCCGGT |
|  | F：TGAGCCGATAGTCCCCCTAA |
| NLRP3 | R：GAGCTGGACCTCAGTGACAATGC |
|  | F：ACCAATGCGAGATCCTGACAACAC |
| AIM2 | R：AGGTTAATGTCCCGCTGA |
|  | F：TCCAAGGGGCTGAGTTT |
| NLRC4 | R：TGAATAAGGGCTCGGCTA |
|  | F：TCCCAAGAAGAGGGCAA |
| β-actin | R：5′-CCTGGCACCCAGCACAAT-3′ |
|  | F：5′-GGGCCGGACTCGTCATAC-3′ |

**Supplementary figure legends**

Figure S1. (A) Representative western blot images showing changes in the levels of caspase 1-p20, gasdermin D (GSDMD-N), interleukin 18 (IL-18), IL-1β cleaved for 5 days after radiation only. (B) Representative western blot images showing changes in the levels of NLRP3, ASC, caspase 1-p20, gasdermin D (GSDMD-N), interleukin 18 (IL-18), IL-1β cleaved on the day following infection treatment in each group.
